# Cerebellar contribution to emotion regulation and its association with medial frontal GABA level

**DOI:** 10.1093/scan/nsae091

**Published:** 2024-12-02

**Authors:** Yumi Oboshi, Toshiki Iwabuchi, Yohei Takata, Tomoyasu Bunai, Yasuomi Ouchi

**Affiliations:** Biofunctional Imaging Laboratory, Division of Preeminent Bioimaging Research, Institute of Photonics Medicine, Hamamatsu University School of Medicine, Chuo-ku, Hamamatsu 431-3192, Japan; Department of Rehabilitation, Gifu University of Health Sciences, Gifu 500-8281, Japan; Research Center for Child Mental Development, Hamamatsu University School of Medicine, Chuo-ku, Hamamatsu 431-3192, Japan; United Graduate School of Child Development, Osaka University, Kanazawa University, Hamamatsu University School of Medicine, Chiba University, and University of Fukui, Suita 565-0871, Japan; Hamamatsu Photonics K.K., Global Strategic Challenge Center, Hamamatsu 434-8601, Japan; Neurology, Hamamatsu University Hospital, Chuo-ku, Hamamatsu 431-3192, Japan; Biofunctional Imaging Laboratory, Division of Preeminent Bioimaging Research, Institute of Photonics Medicine, Hamamatsu University School of Medicine, Chuo-ku, Hamamatsu 431-3192, Japan; United Graduate School of Child Development, Osaka University, Kanazawa University, Hamamatsu University School of Medicine, Chiba University, and University of Fukui, Suita 565-0871, Japan

**Keywords:** emotion regulation, magnetic resonance spectroscopy, functional magnetic resonance imaging, cerebellum, distancing, gamma-aminobutyric acid

## Abstract

As a tactic to regulate emotions, distancing involves changing perspectives to alter the psychological distance from stimuli that elicit emotional reactions. Using magnetic resonance spectroscopy and functional magnetic resonance imaging, this study aimed to examine (i) whether the neural correlates of emotion upregulation via distancing differ across emotional valence (i.e. emotional responses toward positive and negative pictures), and (ii) whether the gamma-aminobutyric acid (GABA) concentration in the medial prefrontal cortex (MPFC), one of the crucial areas of emotion regulation, is correlated with brain activity related to either negative or positive emotion upregulation. Thirty-four healthy Japanese adults participated in this study. Compared to the condition involving positive emotion upregulation, negative emotion upregulation induced increased activation in the MPFC, left temporoparietal junction, bilateral anterior insula, pre-supplementary motor area, and bilateral cerebellum. In contrast, when comparing positive emotion upregulation with negative emotion upregulation, no significant activation was found. Right cerebellar activity during negative emotion upregulation was positively correlated with GABA concentration in the MPFC. These findings provide evidence of cerebellar involvement in the upregulation of negative emotion via distancing and its association with the prefrontal GABA concentration.

## Introduction

Regulating emotional responses to affective experiences is a crucial skill in daily life. Cognitive reappraisal, the reinterpretation of an initial emotional appraisal of a situation, is an effective emotion-regulation strategy (Webb et al. 2012). For example, when presented with a picture that induces positive or negative emotions, one’s emotional responses can be “downregulated” by taking a third-person perspective to detach oneself from the situation. In contrast, imagining one’s direct involvement in a situation will deliberately upregulate the elicited emotion. This tactic for cognitive reappraisal called “distancing” involves changing one’s mental distance from stimuli or situations that induce emotional responses via perspective taking (i.e. decreasing emotions by taking a third-person perspective or increasing emotions by taking a first-person perspective) (Ochsner et al. 2012, Powers and LaBar 2019). For example, emotional responses can be positively upregulated in situations where we share the happiness of close ones as if it were our own and negatively upregulated in situations where close ones are sad, and we feel sad as a result.

Neuroimaging studies have identified the neural underpinnings of cognitive reappraisal, including distancing, during the regulation of affective responses to emotion-inducing stimuli. Most of these functional magnetic resonance imaging (fMRI) studies have targeted the downregulation of negative emotional responses (Diekhof et al. 2011, Buhle et al. 2014, Frank et al. 2014, Morawetz et al. 2017, Pico-Perez et al. 2019, Powers and LaBar 2019). Reducing negative emotional responses to stimuli by distancing modulates activity in regions such as the anterior cingulate cortex (ACC), medial prefrontal cortex (MPFC), dorsolateral and ventrolateral prefrontal cortices (DLPFC and VLPFC), insular cortex, and inferior parietal cortex (Diekhof et al. 2011, Morawetz et al. 2017, Pico-Perez et al. 2019, Powers and LaBar 2019). However, the relevant neural system may depend on emotional valences (positive or negative) and regulation goals (upregulation or downregulation). Regarding possible neural differences between regulation goals, Ochsner et al. (2004) used a distancing strategy in which participants adopted a first- or third-person perspective to upregulate or downregulate negative emotions, respectively. They found a specific activation pattern in the medial prefrontal cortex when negative emotions were downregulated by taking a third-person (i.e. detached or distanced) perspective. No activation was observed during the upregulation of negative emotions by taking a first-person perspective. These results suggest that upregulation and downregulation via distancing may have distinct neural correlates. In contrast to the downregulation of negative emotions through the adoption of a detached third-person perspective, the upregulation of negative emotions through distancing requires the adoption of a first-person perspective. In other words, upregulation via distancing is considered to entail “putting oneself in someone else’s shoes,” possibly resulting in increased activation in the mentalizing system (Spreng et al. 2009).

In addition, given the paucity of relevant studies, the present study addressed whether different neural systems are recruited for different emotional valences during emotion upregulation by distancing. Seo et al. (2014) reported that the ventromedial prefrontal cortex and amygdala were more activated by negative than positive emotional stimuli during active engagement in emotion downregulation; however, emotion upregulation was not examined in that study. Two recent fMRI studies have adopted both emotional valences (positive and negative) and regulatory goals (upregulation and downregulation) in their experimental designs (Min et al. 2022, Sokolowski et al. 2022). However, Sokolowski et al. (2022) investigated the differences in brain activation patterns between the positive and the negative emotional valence, regardless of the regulatory goals; they did not examine the effect of stimulus valence in a goal-specific manner. Min et al. (2022) stated that they found few activation differences between the positive and the negative valence during emotion upregulation. However, whether the same applies to neural correlates of distancing is unclear because not all participants in their study reported using this strategy. Only one study has reported significant differences across valences during emotion upregulation via distancing (Kim and Hamann 2007), to the best of our knowledge. According to Kim and Hamann (2007), all participants reported that they imagined the depicted scenes to be personally relevant to themselves to increase their emotional responses toward positive or negative pictures. In other words, all participants used distancing as their strategy for upregulation. However, despite the seminal value of Kim and Hamann’s study, their findings were obtained from extremely small samples (*N* = 10) in terms of the standard of recent neuroimaging studies. Therefore, comparing the neural correlates of distancing across emotional valences using a larger sample size would be worthwhile. The present study focused on the neural correlates of upregulation, which have been much less studied than those of downregulation, and their differences between the positive and the negative emotional valence. Here, using images whose emotional valence and arousal levels have been previously investigated as positive and negative stimuli (Lang et al. 2008), we assume that positive stimuli elicit positive responses and that negative stimuli elicit negative responses.

Another important issue in recent research on emotion regulation has been the role of gamma-aminobutyric acid (GABA), a primary inhibitory neurotransmitter. Deficits in the GABAergic system are associated with various psychiatric disorders (Schur et al. 2016, Simmonite et al. 2022), for example, and problems in emotion regulation may be a common transdiagnostic factor in the pathophysiological mechanisms underlying these disorders (Gross and Jazaieri 2014, Aldao et al. 2016). In contrast, studies on healthy individuals have shown a relationship between brain GABA levels and brain function during cognitive processing. Previous studies using fMRI and proton magnetic resonance spectroscopy (^1^H-MRS) have suggested possible links between GABA concentration and blood oxygen level-dependent (BOLD) responses during various cognitive tasks (see Kiemes et al. 2021 for a review). Regarding emotional processing, Northoff et al. (2007) found an association between increased GABA levels in the ACC/MPFC and negative BOLD responses in the same region while viewing emotional pictures. Given that the MPFC is considered a part of the core system for emotion regulation through distancing (Powers and LaBar 2019), GABA levels in this region can be hypothesized to be associated with brain activation during emotion regulation. The Mescher–Garwood point-resolved spectroscopy sequence (MEGA-PRESS) allows researchers to quantify macroscopic GABA concentration *in vivo* (Mescher et al. 1998). Some studies have investigated the relationship between GABA concentration in emotion-related regions such as the ACC and fMRI activation during engagement in emotion-related tasks (Levar et al. 2017a, 2017b). However, the possible associations between GABA concentration and BOLD responses related to emotion regulation by distancing have not yet been investigated.

Given this background, this study had two purposes. We aimed to (i) investigate whether the neural activation patterns during emotion upregulation by distancing differ between emotional valences (positive or negative) and (ii) post-hoc examine whether such activations are associated with GABA concentration in the ACC/MPFC, one of the regions consistently implicated in emotion regulation processes. Additionally, we explored possible sex differences in the observed fMRI activation or GABA concentration. Previous studies have suggested possible biological sex differences in the cortical GABAergic systems (Cosgrove et al. 2007, Pandya et al. 2019), but only a few MRS studies have investigated sex differences in GABA levels using MRS. For example, Sanacora et al. (1999) reported increased occipital GABA levels in females compared to males, but it may be important to provide new sex comparison data for future syntheses such as meta-analyses. Moreover, neural correlates of emotion regulation might differ between the biological sexes (McRae et al. 2008, Mak et al. 2009, Domes et al. 2010, Min et al. 2023). However, the results of these studies are inconclusive. Some of these studies have suggested higher activity in female participants compared to male participants during active emotion regulation (McRae et al. 2008, Min et al. 2023), whereas other studies reported the opposite pattern (Mak et al. 2009, Domes et al. 2010). Therefore, the aim of our exploratory analysis was to provide new evidence on this topic.

## Materials and methods

### Participants

Thirty-four healthy Japanese adults (18 females; 20–25 years; mean age, 22.4; SD, 1.6) were enrolled. Data from one participant (21 years, female) were excluded from the analysis because of incomplete fMRI data acquisition owing to technical problems. None of the participants reported a history of neuropsychiatric disorders, and all participants had normal or corrected-to-normal vision. The study was approved by the Ethics Committee of Hamamatsu University School of Medicine and conducted in accordance with the Declaration of Helsinki. Each participant provided written informed consent.

### Stimuli

We used 20 positive and 20 negative pictures selected from the International Affective Picture System (IAPS) for the emotion regulation task (Lang et al. 2008; see Supplementary data for further details). Because we directly compared the upregulation of positive and negative emotions, neutral pictures are not included in the present study.

### Task procedure

**Figure 1.**
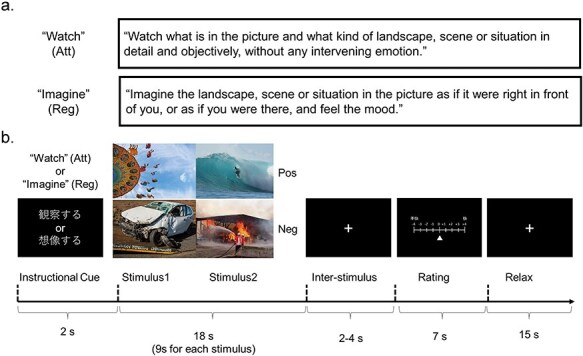
Experimental task. (A) Instructional content of each of the attention (Att) and regulation (Reg) conditions. (B) Schematic representation of each trial. After the first instructional cue, two pictures were presented. During the picture presentation, participants were instructed to either upregulate their emotional responses or to simply look at the pictures attentively. Participants were then asked to rate their emotional state. We replaced the example images that were used with similar images because of the copyright restriction of the International Affective Picture System (top left, an image by Scott Webb via Pixabay under the Creative Commons Zero (CC0) license https://creativecommons.org/public-domain/cc0/; top right, an image by Andy Perdana via Pixabay under the Creative Commons Zero (CC0) license https://creativecommons.org/public-domain/cc0/; bottom left, an image by Steve Buissinne via Pixabay under the Creative Commons Zero (CC0) license https://creativecommons.org/public-domain/cc0/; bottom right, an image by Dirk (Beeki®) Schumacher via Pixabay under the Creative Commons Zero (CC0) license https://creativecommons.org/public-domain/cc0/). Att, attention condition; Reg, regulation condition; Pos, positive condition; Neg, negative condition.

In each trial of the cognitive reappraisal task, an instructional cue [“Imagine” for the regulation (Reg) condition or “Watch” for the attention (Att) condition in Japanese] was presented for 2 s (Fig. 1). Two pictures were then consecutively presented for 9 s each. In the Reg condition, the participants were requested to upregulate their positive or negative emotional reactions during the picture presentation by imagining themselves as directly involved in the situations being depicted. Alternatively, in the Att condition, participants were requested to attentively watch the pictures presented. After an interval ranging from 2 to 4 s (3 s on average), where a fixation cross was shown at the center of the monitor, a rating scale was presented for 7 s. Within this period, we instructed participants to rate their current emotional state on a scale that ranged from −4 (“unpleasant”) to 4 (“pleasant”) by pressing buttons with their right middle or index finger to move the cursor rightward (+1) or leftward (−1), respectively. A fixation cross was presented for 15 s at the end of each trial.

The experiment consisted of four runs of fMRI scanning. In each run, either positive (Pos) or negative (Neg) stimuli were selectively presented. Each run consisted of 10 trials. Half of the trials were assigned to the Reg conditions, while the other half were assigned to the Att conditions. Thus, in total, four conditions (PosReg, PosAtt, NegReg, and NegAtt) were used in the experiment. Further details on the given instruction, practice sessions, and stimulus presentation are provided in the Supplementary data.

### fMRI data acquisition

A 3-Tesla MR scanner (Ingenia, Royal Philips, Eindhoven, The Netherlands) was used to obtain functional and anatomical MRI data (see Supplementary data for parameter information).

### ^1^H-MRS data acquisition

The ^1^H-MRS data were acquired using MEGA-PRESS (Supplementary data). The ACC/MPFC ROI (voxel size, 20 mm × 30 mm × 30 mm) was placed in front of the anterior boundary of the genu of the corpus callosum (Supplementary Fig. S1).

### Behavioral data analysis

For the participants’ mean rating scores, a two-way repeated-measures analysis of variance (ANOVA) was conducted with within-subject factors associated with the valence (Pos, Neg) and task condition (Reg, Att).

### fMRI data analysis

We preprocessed the fMRI data using the SPM12 (available at http://www.fil.ion.ucl.ac.uk/spm/). After preprocessing, we estimated the voxel-wise condition effects for each participant using a general linear model approach. See Supplementary data for further detailed descriptions.

We subsequently submitted the four conditional beta maps (PosReg, PosAtt, NegReg, or NegAtt) obtained from the first-level analysis to a full factorial ANOVA with two within-subject factors of the task condition (Reg and Att) and valence (Pos and Neg) for the second-level random-effects analysis. We conducted *t*-tests to identify the effects of interest. The initial threshold was set at *P* < .001 (uncorrected for multiple comparisons at the peak level). Then, the statistical significance for inference was thresholded at *P* < .05 (family-wise error [FWE] corrected at the cluster level). We compared the NegReg and PosReg conditions (i.e. PosReg > NegReg and NegReg > PosReg) to examine the valence effects on brain activity associated with emotion upregulation. Based on previous studies (Hayes et al. 2013, Shibata et al. 2013), the activation map for the NegReg > PosReg contrast was exclusively masked by the NegAtt > PosAtt contrast (*P* < .05, uncorrected for multiple testing) to exclude the effects of stimulus differences across valences. Additionally, we examined the reverse contrast (PosReg > NegReg) with an exclusive mask using the contrast of PosAtt > NegAtt (*P* < .05, uncorrected for multiple comparisons) to identify the regions uniquely associated with positive emotion upregulation. To ensure the robustness of this analysis, in addition to performing the group analysis with an exclusive masking, we directly compared the NegReg > PosReg contrast with the NegAtt > PosAtt contrast using a paired *t*-test (see Supplementary data).

### ^1^H-MRS data quantification

Using the spectral registration method implemented in Gannet 3.1, frequency- and phase-drift correction was performed (Near et al. 2015). The corrected MRS data were then fitted in LCModel (Version 6.3-1 N) using a built-in simulated basis set to quantify water-scaled concentrations of metabolites, including GABA + macromolecules (hereafter referred to as GABA+) and N-acetylaspartate (NAA). We excluded spectral data with Cramer–Rao lower bounds of >20% from further analysis. We then calculated the relative GABA+ concentration as a ratio to NAA (hereafter denoted as GABA/NAA). GABA/NAA ratios were used as an index of GABA levels in the ACC/MPFC.

### Region of interest analyses

Spherical region of interests (ROIs) with a 6-mm radius were created using the peak coordinates for the contrast of emotion upregulation (e.g. NegReg > PosReg exclusively masked by NegAtt > PosAtt) as centers. Individual beta values for four experimental conditions were extracted from the ROIs. MarsBar (Brett et al. 2002) was used for ROI creation and data extraction.

### Correlational analysis

We calculated Pearson’s correlation coefficient between GABA/NAA ratios and NegReg minus PosReg differences in brain activity (beta value) for each functional ROI to explore the association between GABA concentration in the ACC/MPFC and brain activity related to emotion upregulation. We evaluated the statistical significance of the correlation coefficients by performing permutation tests with 50 000 times random assignment of participants to the differences in beta values. Given the exploratory nature of this analysis, we used false discovery rate (FDR)-corrected *q* < 0.1 as a threshold. For the regions whose activity was significantly associated with GABA concentration, we calculated Pearson’s correlation coefficient between upregulation-related brain activity (e.g. NegReg minus PosReg differences in beta values) and the individual efficacy in emotion regulation. The difference in the rating scores (NegReg minus PosReg) was used as an index of the efficacy of a participant’s emotional response upregulation, assuming that the more effectively a participant upregulate their emotion, the lower and higher the emotion rating scores will be in the negative and positive upregulation conditions, respectively.

### Exploratory analysis

Using unpaired *t*-test for each ROI and additional group-level analysis, post-hoc exploratory analyses were conducted to examine sex differences in either brain activity related to emotion regulation or GABA concentration in the ACC/MPFC (Supplementary data).

## Results

### Behavioral data

Table 1 presents the emotion rating scores. Two-way repeated-measures ANOVA revealed that the interaction between task condition and valence and the main effect of valence were significant (valence, *F*[1,32] = 312.2, *P* < .001; task condition, *F*[1,32] = 1.9, *P* = .18; valence × task interaction, *F*[1,32] = 58.4, *P* < .001). A post-hoc analysis showed that the simple main effect of valence was significant in the Reg and Att conditions (Reg, *F*[1,64] = 370.6, *P* < .001; Att, *F*[1,64] = 173.2, *P* < .001). The negative stimuli had significantly lower emotion rating scores than the positive stimuli in both Reg and Att conditions. We also found a significant simple main effect of task condition on the Pos and Neg stimuli (Pos, *F*[1,64] = 56.7, *P* < .001; Neg, *F*[1,64] = 41.0, *P* < .001). This suggests that rating scores were significantly lower in the Reg condition than in the Att condition for negative stimuli, and vice versa for positive stimuli. Altogether, the behavioral results suggest that the participants successfully upregulated their emotions to positive and negative affective pictures.

**Table 1.** Self-reported rating scores for emotional states

	PosReg	PosAtt	NegReg	NegAtt
Mean	2.44	1.57	−2.65	−1.91
SE	0.12	0.15	0.12	0.14

PosReg, positive regulation condition; PosAtt, positive attention condition; NegReg, negative regulation condition; NegAtt, negative attention condition; SE, standard error.

### Neuroimaging data

**Figure 2.**
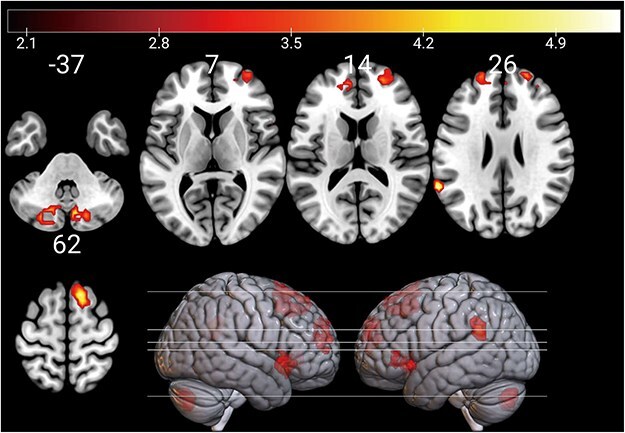
Brain activation in the negative regulation condition compared to the positive regulation condition. The color bar denotes *t*-values.

**Figure 3.**
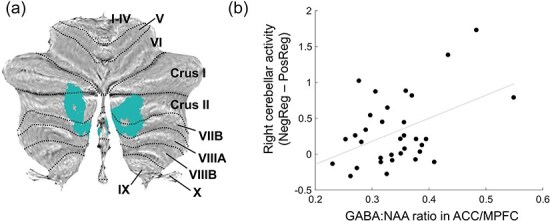
Association between GABA concentration in the ACC/MPFC and activation in the right cerebellum. (a) Colored regions show the bilateral cerebellar clusters that were markedly activated when comparing the negative upregulation with the positive upregulation conditions. These clusters primarily overlap with Crus II. (b) GABA concentration in the ACC/MPFC was positively correlated with the right cerebellar activity. NegReg, negative regulation condition; PosReg, positive regulation condition.

Contrasting the NegReg condition with the PosReg condition revealed significant activation in the bilateral anterior insula (aIns), left temporoparietal junction (TPJ) extending from the left supramarginal gyrus to the superior temporal cortex, pre-supplementary motor area (pre-SMA), ACC/MPFC, and bilateral cerebellum (see Fig. 2 and Table 2). We used the spatially unbiased atlas template of the cerebellum and brainstem (SUIT) toolbox to identify the spatial cerebellar subregions and visualize the cluster locations on a flat cerebellar map (Diedrichsen 2006, Diedrichsen et al. 2009, 2011, 2015). Most parts of the bilateral cerebellar clusters overlapped with Crus II (Fig. 3a, with the peak coordinate for the right cluster [9, −73, −37] and that for the left cluster [−18, −73, −40] both falling within Crus II). For the contrast of the PosReg condition versus the NegReg condition, on the other hand, we found no significant activation. Direct comparison of the NegReg > PosReg contrast with the NegAtt > PosAtt contrast failed to detect significant activations in the right cerebellum, left TPJ, and bilateral aIns (see Supplementary data  Supplementary Table S1). When using a more liberal threshold (*P* < .005, uncorrected at the peak level, and *P* < .05, FWE corrected at the cluster level), we found a significant cluster encompassing the right cerebellum and the inferior occipital cortex (see Supplementary Fig. S2). For reference, fMRI results for other contrasts are provided in Supplementary Table S2.

**Table 2.** Activated regions for comparisons between the positive and negative valences during emotion regulation

		MNI				
Anatomical label	Side	*x*	*y*	*z*	*k*	*t* value
PosReg > NegReg (exclusively masked by the contrast of PosAtt > NegAtt)						
n.s.						
NegReg > PosReg (exclusively masked by the contrast of NegAtt > PosAtt)						
aIns	L	−33	23	−7	120	5.33
aIns/TP	R	48	17	−10	186	5.29
SMG	L	−60	−49	26	61	5.12
pre-SMA	R/L	12	20	62	229	5.07
Cerebellum	L	−18	−73	−40	88	4.68
Cerebellum	R	9	−73	−37	78	4.67
ACC/MPFC	L/R	−9	50	14	221	4.32

*P* < .05, family-wise error corrected for multiple comparisons at the cluster level. Cluster sizes (*k*) and voxel-level *t* values are shown. MNI, Montreal Neurological Institute; n.s., not significant; R, right; L, left; aIns, anterior insula; TP, temporal pole; SMG, supramarginal gyrus; pre-SMA, pre-supplementary motor area.

### Correlational analysis

**Figure 4.**
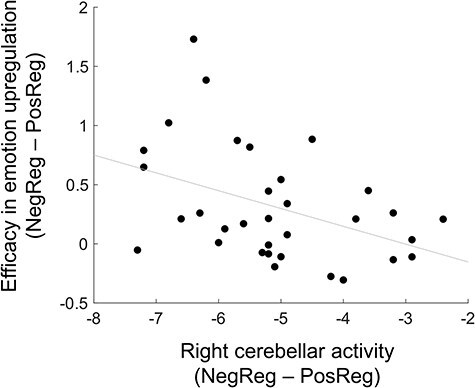
Correlation between the right cerebellar activity and emotion upregulation efficacy. The *x*-axis shows the difference in beta values (unitless) between the NegReg and PosReg conditions, while the *y*-axis represents the difference in emotion rating scores between the NegReg and PosReg conditions. NegReg, negative regulation condition; PosReg, positive regulation condition.

The GABA/NAA ratio in the ACC/MPFC was significantly correlated with right cerebellar activity (Fig. 3b; *r* = 0.44, *P* = .008, FDR-corrected *q* = 0.056 < 0.1, permutation test). For the other functional ROIs, no significant association was found between the GABA/NAA ratio and brain activity after FDR-correction for multiple testing (left aIns, *r* = 0.11, *P* = .27; right aIns, *r* = 0.24, *P* = .09; left SMG, *r* = 0.21, *P* = .12; pre-SMA, *r* = 0.08, *P* = .32; left cerebellum, *r* = 0.33, *P* = .03; and ACC/MPFC, *r* = 0.03, *P* = .43). Right cerebellar activity was significantly correlated with emotion regulation efficacy (the NegReg minus PosReg difference in rating scores; Fig. 4; *r* = −0.41, *P* = .006, permutation test).

### Exploratory analysis

GABA concentration in the ACC/MPFC ROI did not differ between males and females (*t* = 0.85, *P* = .39). No significant sex differences were observed in the activity of any ROI (Supplementary Table S3). The additional group-level fMRI analysis revealed significant activation in the left supramarginal gyrus for the contrast of (NegReg/male > PosReg/male) > (NegReg/female > PosReg/female) (Supplementary Fig. S3). For the other contrasts, no significant activation was found.

## Discussion

The present MRS-fMRI study investigated BOLD responses to distancing-based upregulation of positive and negative emotions. Moreover, we examined the association between GABA levels in the ACC/MPFC and these BOLD responses. Negative emotion upregulation induced significant activation in some regions that have been associated with cognitive reappraisal (the bilateral insula, pre-SMA, left TPJ, and ACC/DMPFC). Furthermore, the bilateral cerebellum was also activated for negative emotion upregulation. We subsequently found that the GABA levels in the ACC/MPFC were positively correlated with right cerebellar activity for negative emotion upregulation. Additionally, we found a significant positive correlation between right cerebellar activity (related to negative emotion upregulation) and emotion regulation efficacy in the task.

Our findings highlight the importance of the right cerebellum in upregulating negative emotions via distancing. This is in line with the evidence of the relationship between emotional processing and the cerebellum. For example, neuropsychological studies have reported that cerebellar damage can cause emotional problems (Schmahmann and Sherman 1998, Schmahmann et al. 2007). Studies using brain stimulation have also suggested an association between the cerebellum and emotional processing (Schutter et al. 2009, Schutter and van Honk 2009, Ferrucci et al. 2012, Ferrari et al. 2018, 2022). However, reports of cerebellar activation during emotion regulation tasks are relatively scarce. The inconsistency between the present study and previous fMRI studies may have resulted from different experimental paradigms. As stated in the “Introduction” section, only Kim and Hamann (2007) reported brain activation differences during distancing between positive and negative upregulations. Additionally, previous studies may have underestimated the cerebellar involvement in emotion regulation, as pointed out by Van Overwalle et al. (2020a) and Frazier et al. (2022). The relationship between emotion and the cerebellum has only been reported by neuroimaging rather recently, and some older studies may not have covered the cerebellum during scans.

This study demonstrated successive associations among GABA concentration in the ACC/MPFC, right cerebellar activity, and emotion regulation efficacy. This is consistent with previously demonstrated associations between altered GABA concentration in the ACC/MPFC and mood disorders such as depression or bipolar disorder (Simmonite et al. 2022). Although our ACC/MPFC ROI was spatially distant from the cerebellum, resting-state fMRI studies have shown functional connectivity between the medial prefrontal area and the cerebellum in the healthy brain (Buckner et al. 2011, Guo et al. 2014, Metoki et al. 2022). Moreover, the medial prefrontal area and the cerebellum (specifically Crus II) were functionally coupled with each other during social cognition tasks (Van Overwalle et al. 2015, Van Overwalle and Marien 2016). Additionally, atypical functional connectivity between the MPFC and the posterior part of the cerebellum, including Crus II, has been shown in patients with mood disorders such as bipolar disorder (Chen et al. 2019) and depression (Alalade et al. 2011, Ma et al. 2013). Given the known association between regional GABA concentration and BOLD responses during cognitive tasks (Kiemes et al. 2021), such mediofrontal–cerebellar coupling, which is possibly important for typical emotion regulation functioning, could be mediated by GABAergic function in the medial frontal lobe. Of note, left cerebellar activity was also activated for the NegReg > PosReg contrast and was weakly associated with ACC/MPFC GABA levels (*r* = 0.33, *P* = .03 uncorrected), although the correlation did not survive multiple comparisons correction. Thus, we must be cautious in concluding that the association with GABA is specific to the right but not the left cerebellum.

The bilateral aIns was more activated to upregulate negative emotions than positive emotions. The insula is crucial for emotion processing, and its involvement in emotion regulation has been corroborated by previous studies. This study provides novel insight into negative emotion upregulation, which has not been well-studied before. In contrast, the present study found no significant activation in the amygdala, which is another essential processing center for emotion (Adolphs 2008, Pessoa and Adolphs 2010). This seems inconsistent with the results of repeated reports that describe the amygdala activity to be modulated by regulation of affective responses (Diekhof et al. 2011, Buhle et al. 2014, Frank et al. 2014). Although the aIns and amygdala have been consistently implicated in negative emotional reactions to stimuli, the precise roles of these regions in emotion regulation are somewhat arguable. This lack of activation in the amygdala may have subtle implications for the distinct roles of the amygdala and the aIns in processing negative emotions. An fMRI meta-analysis reported that the aIns is preferentially activated for empathic reactions to others’ pain, whereas the amygdala activation is selective for reactions to emotional faces (Ding et al. 2020). Another line of evidence, although controversial (Tippett et al. 2018), suggests the specificity of the amygdala and the aIns in processing fear and disgust, respectively (Fusar-Poli et al. 2009). Further studies involving careful manipulation of experimental stimuli are needed to disentangle the associations between these emotion-related regions and emotion regulation.

Some regions involved in negative emotion upregulation, especially the left TPJ and ACC/MPFC, roughly overlapped with the so-called default mode network, which may be relevant to “self-projection.” Self-projection is the ability to project oneself from one’s present experience into alternative temporal, spatial, and/or mental perspectives (Buckner and Carroll 2007), and thus this function is essentially required for the emotion regulation tactic used in the present study. Using self-projection is also involved in the mentalizing process (i.e. estimating others’ mental states), and available evidence supports that a common set of regions, including the MPFC and TPJ, underlies the mentalizing process and other types of self-projection (e.g. imagining the future) (Spreng et al. 2009).

However, it should be noted that the left TPJ and the bilateral aIns were not found to be activated in the direct comparison of the NegReg > PosReg contrast with the NegAtt > PosAtt contrast. While these regions may play some role in the upregulation of negative emotions, cautious interpretation is required. In particular, the left inferior parietal region was also activated in the PosReg > PosAtt comparison (see Supplementary Table S2). Therefore, in contrast to the bilateral aIns, the left TPJ may not only be associated with negative upregulation but also with positive upregulation.

Interestingly, according to a recent meta-analysis, the cerebellar cluster that we found also fell into Crus II of the cerebellum, which is involved in mentalizing (Van Overwalle et al. 2020a, 2020b). Participants in the present study were explicitly asked to view the pictures as if they were directly involved in the situation; in other words, they were asked to “put themselves in someone else’s shoes” in the Reg conditions. Thus, it seems reasonable to assume that the mentalizing system (or more broadly, the self-projection system) was recruited to upregulate negative emotional responses to these pictures. The degree to which the self-projection system is involved in emotion regulation may be influenced by how participants are instructed about the strategies; this is an interesting topic for future studies. Additionally, whether mentalizing is based on exactly the same neural architecture as other domains of self-projection, including episodic memory, thinking about the future, and spatial navigation, is controversial (Immel et al. 2022); however, this is beyond the scope of this study. How an individual’s empathic accuracy modulates activation in the self-projection system for distancing-based emotion regulation would be another interesting research avenue to pursue.

The present study also compared the PosReg condition to the NegReg condition but found no significant increase in any region for the PosReg condition. This result contradicts the findings of Kim and Hamann (2007), who found a reverse activation pattern. They reported that broad areas in the prefrontal cortex and left amygdala were more activated for positive upregulation than for negative upregulation. One possible explanation for this is that the effect of upregulating positive emotions in the present study was too small to be detected. As indicated in some previous studies, common regions may be involved in emotion upregulation regardless of the valence. If this is the case, our finding of elevated brain activity for negative rather than positive upregulation may imply that our negative stimuli were stronger in terms of inducing emotional reactions than the positive stimuli. Based on the results of this study, we cannot draw any certain conclusions regarding positive emotion upregulation.

An additional whole-brain analysis detected an increased activation of a subregion in the left supramarginal gyrus in males compared to that in females when the NegReg condition was compared with the PosReg condition. This subregion was adjacent to the left TPJ cluster, as observed in the main analysis. Regarding the activated clusters in the main whole-brain analysis, we found no significant sex differences in the activity related to negative upregulation. These results suggest that a broader area of the left TPJ is involved in negative emotion upregulation in males than in females. This result seems contradictory to the finding that females have a greater neural cost in upregulating emotional responses to negative images (Gardener et al. 2013). Although accumulating evidence suggests sex differences in the neural correlates of emotion regulation, the results are mixed; for example, Domes et al. (2010) reported increased prefrontal activity in males than in females, while McRae et al. (2008) demonstrated the opposite patterns. The issue of sex differences should be further investigated by future studies with larger samples.

This study had some limitations. First, due to the small sample size and exploratory nature of the study, a relatively lenient statistical threshold was adopted for multiple comparisons correction in the correlational analysis. In particular, the exploratory analysis of sex differences presented here is intended for reference only. In addition, the right cerebellar activity was confirmed by the direct comparison of the NegReg > PosReg contrast with the NegAtt > PosAtt contrast only when a liberal threshold was applied. Further studies are required to confirm the generalizability of our findings. Second, although a significant correlation was observed between the efficacy of regulating emotional responses and right cerebellar activation, the participants’ emotional states were evaluated based solely on their subjective ratings. Future studies using physiological measures (e.g. skin conductance response) are required. Third, this study used stimuli from the IAPS database; however, the people in these pictures were generally not Asian. Given the cross-race effect of face perception (Young et al. 2012), changing characteristics of the stimuli may have had some impact on our findings. However, we believe that the impact may not be crucial because of careful control across conditions. Fourth, the technical limitations of MRS limited the regional specificity of GABA measurement. We must wait for technical advances in non-invasive measurement of brain metabolites to resolve this issue. Finally, the present study did not focus on any specific aspect of distancing strategy (e.g. spatial, temporal, objective, or hypothetical distancing; Powers and LaBar 2019). Given the content of the given instruction, it is likely to be closer to a temporal and spatial distancing strategy, but future research should clarify this point.

In conclusion, this study is the first to report an association between GABA concentration in the ACC/MPFC and right cerebellar activation for negative emotion upregulation via distancing. Further studies on the cerebellar role in emotion regulation and the link between the GABAergic system and emotion regulation are warranted.

## Supplementary Material

nsae091_Supp

## Data Availability

The dataset analyzed in this study is available from the corresponding author upon reasonable request.
