# Supplementary material for: Cerebellar contribution to emotion regulation and its association with medial frontal GABA level
Source: Soc Cogn Affect Neurosci. 2024 Dec 2;20(1):nsae091. doi: 10.1093/scan/nsae091 (PMC11776713; doi:10.1093/scan/nsae091)
Supplement: nsae091_Supp [file nsae091_supp.zip › nsae091_Supp/scan-23-219-File008.docx]

**Supplementary Methods**

*Stimuli*

As mentioned in the main text, 20 positive and 20 negative pictures were selected from the International Affective Picture System (IAPS) image set. The following numbers were selected as the positive pictures: 2216, 5260, 5829, 5833, 7492, 7502, 8163, 8170, 8370, 8496, 5210, 5600, 5623, 5700, 5910, 8190, 8210, 8420, 8490, and 8492. The following images were used for the negative pictures: 3530, 5972, 7359, 9571, 9592, 9600, 9901, 9909, 9911, 9941, 1111, 1201, 9163, 9428, 9611, 9622, 9623, 9900, 9908, and 9920. The mean normative ratings of valence (pleasantness) and arousal for positive pictures were 7.60 and 5.82 (SD = 0.30 and 0.68), respectively, whereas those for negative pictures were 2.70 and 5.95 (SD = 0.54 and 0.47), respectively. Each stimulus picture was assigned to one of the two task conditions: the regulation (Reg) or attention (Att) conditions. Each participant was presented with each picture twice in the experiment, once in the Reg condition and once in the Att condition.

*Task procedure*

In the upregulation condition, participants were instructed to imagine the situation in the presented picture vividly, as if they were there, right in front of them, and to feel the mood; in the attention condition, participants were instructed to observe the situation objectively and in detail.

Approximately one to two weeks before the fMRI experiment, the same participants as those who participated in the fMRI experiment performed the same task outside the scanner (pre-training session). In this pre-training session, most participants scored lower and higher in the upregulation conditions for the negative and positive stimuli, respectively, than in the attention conditions for the negative and positive stimuli. Most participants also rated positive stimuli higher than negative stimuli. These pre-training results confirmed the effectiveness of the emotion regulation task. Of note, a different set of images was used in this pre-training session than that used in the fMRI experiment. There were no significant differences in emotional valence and arousal levels between the image sets for the pre-training and fMRI sessions. In addition, a shortened practice session outside the scanner was performed immediately before each fMRI experiment to confirm that participants understood the instructions. All images used in this practice run were different from those used in the main measurement session (both the valence and arousal levels were not significantly different from those in the main measurement session).

For the fMRI experiment, the order of the four experimental runs was either Neg-Pos-Neg-Pos or Pos-Neg-Pos-Neg. Across participants, the order was counterbalanced. In each run, the order of the task conditions (Reg or Att) was pseudorandomized for each participant. The ratio of which condition (either the Reg or Att condition) preceded the other in an fMRI run was balanced for each participant. We used the Presentation software (Neurobehavioral Systems, Inc., Albany, CA, USA; <https://www.neurobs.com/>) to control stimulus presentation. The participants viewed the stimuli through a head-mounted display that can be used in MRI scanners (Visual Stim Digital; Resonance Technology Company, Inc., Los Angeles, USA).

After the fMRI experiment, the participants were asked to view the picture stimuli used in the experiment outside the scanner and to rate how aroused they had felt for each stimulus during the fMRI scan using a visual analogue scale (VAS) (0: very sleepy, 100: very awake). The results showed that the arousal level was slightly higher for the positive pictures than for the negative pictures (mean arousal score for the positive stimuli, 69.75 ± 11.62; mean arousal score for the negative stimuli, 66.19 ± 13.18; *df* = 32, *t* = -2.11, *p* = 0.043).

*fMRI data acquisition*

To measure BOLD responses as a proxy for brain activity during the cognitive reappraisal task, T2*-weighted gradient-echo echo-planar images were acquired with the following parameters: TR = 2500 ms, TE = 25 ms, flip angle = 90, slice thickness = 3 mm with a 0.5-mm gap, voxel size = 3 mm × 3 mm × 3.5 mm, matrix = 64 × 64, 42 transversal slices in the ascending order, and 184 volumes per run. Additionally, T1-weighted images were collected for normalization of the functional data (TR = 6.1 ms, TE = 2.8 ms, flip angle = 8, voxel size = 1 mm × 1 mm × 1 mm, and 210 sagittal slices).

*^1^H-MRS data acquisition*

The following parameters were used to acquire the MEGA-PRESS MRS data: TR = 2000 ms, TE = 68 ms, 14-ms editing pulses at 1.90/7.46 [on/off] ppm, 2048 complex data points, 160 averages, 2-kHz spectral width, and MOIST water suppression. Participants were at rest during the MRS acquisition.

*fMRI data analysis: preprocessing and first-level analysis*

The fMRI data were preprocessed as follows: Functional images were realigned to the mean image after the first four functional volumes were discarded to allow for T1 equilibrium. Differences in slice acquisition timing were corrected using the middle slice as the reference. After co-registration to the individuals’ anatomical images, the functional images were spatially normalized to the East Asian brain template in two steps: 1) segmentation to estimate normalization parameters and 2) normalization of the functional images with those parameters. Resampled into 3 mm × 3 mm × 3 mm voxels with the seventh-degree B-spline interpolation, the anatomical and functional images were transformed into the Montreal Neurological Institute (MNI) standardized space. Finally, the normalized functional images were smoothed with a 6-mm full width at half maximum (FWHM) Gaussian kernel.

We then conducted a first-level fMRI analysis. Stimulus presentation periods, including cue presentation (20 s) and affective rating periods (7 s) for each experimental condition (PosReg, PosAtt, NegReg, or NegAtt), were separately modeled with box-car functions and then convolved with a hemodynamic response function to create regressors in the design matrix. Six motion parameters were included in the design matrix as covariates to account for movement-related variance. Low-frequency noise was removed using a high-pass filter with a cut-off period of 128 s. Temporal correlations in the fMRI time series were estimated using an autoregressive AR(1) model, which was used to correct for non-sphericity during statistical inference.

*fMRI analysis: direct comparison between the NegReg > PosReg and the NegAtt > PosAtt contrasts*

In the main analysis, we contrasted the NegReg and PosReg conditions with an exclusive masking of the NegAtt > PosAtt contrast (p < 0.05, uncorrected) to exclude contamination of the stimulus difference between the Pos and Neg conditions. However, this analysis did not examine statistically different activation levels between the NegReg > PosReg contrast and the NegAtt > PosAtt contrast. We performed an additional paired t-test to directly examine the statistical differences between these two contrasts. In the first-level analysis, we obtained the contrast images for the NegReg > PosReg and the NegAtt > PosAtt. These two contrast images were then submitted to the group-level paired t-test. In this group-level analysis, we set two contrasts to examine both the [(NegReg > PosReg) > (NegAtt > PosAtt)] and the [(PosReg > NegReg) > (PosAtt > NegAtt)].

*Exploratory analysis*

We performed an unpaired *t*-test to examine whether a sex difference existed in the GABA concentrations and beta values (NegReg minus PosReg) of each functional spherical ROI.

To investigate sex differences, we additionally created a group-level fMRI model with three factors (biological sex, task conditions [Reg, Att], and valence [Pos, Neg]). Four contrasts were tested to examine the sex differences in activation for the NegReg and PosReg contrast (i.e., [NegReg/male > PosReg/male] > [NegReg/female > PosReg/female] or [NegReg/female > PosReg/female] > [NegReg/male > PosReg/male] or [PosReg/male > NegReg/male] > [PosReg/female > NegReg/female] or [PosReg/female > NegReg/female] > [PosReg/male > NegReg/male]). The statistical threshold was set at p < 0.001 (uncorrected for multiple comparisons at the voxel level) and p < 0.05, with family-wise error correction at the cluster level. As in the main fMRI analysis, the activation map of either NegAtt > PosAtt or PosAtt > NegAtt (p < 0.05 uncorrected for multiple comparisons) was exclusively masked to exclude the effect of stimulus differences between valences.
