# Supplementary material for: Cerebellar contribution to emotion regulation and its association with medial frontal GABA level
Source: Soc Cogn Affect Neurosci. 2024 Dec 2;20(1):nsae091. doi: 10.1093/scan/nsae091 (PMC11776713; doi:10.1093/scan/nsae091)
Supplement: nsae091_Supp [file nsae091_supp.zip › nsae091_Supp/scan-23-219-File009.docx]

**Supplementary Results**

*fMRI analysis: direct comparison between the NegReg > PosReg and the NegAtt > PosAtt contrasts*

Using the same threshold as that in the main analysis (i.e., *p* < 0.001, uncorrected at the peak level, and *p* < 0.05, family-wise error [FWE] corrected at the cluster level), three clusters were identified by the [(NegReg > PosReg) > (NegAtt > PosAtt)] contrast. These clusters included the left cerebellum–inferior occipital cortex, the right inferior occipital cortex, and the medial prefrontal cortex/supplementary motor area (Supplementary Table S1). The right cerebellum, which was identified and focused on in the main analysis, was not included in these activated clusters. Only when a more liberal threshold (*p* < 0.005, uncorrected at the peak level, and *p* < 0.05, FWE-corrected at the cluster level) was applied, the right cerebellar activity was identified within the significantly activated clusters (Supplementary Figure S1). The bilateral anterior insula and the left temporoparietal junction were not activated in this analysis.

The contrast of [(PosReg > NegReg) > (PosAtt > NegAtt)] revealed no significant activation even when a liberal threshold (*p* < 0.005, uncorrected at the peak level and *p* < 0.05, FWE-corrected at the cluster level) was used.
