# Supplementary material for: Cerebellar contribution to emotion regulation and its association with medial frontal GABA level
Source: Soc Cogn Affect Neurosci. 2024 Dec 2;20(1):nsae091. doi: 10.1093/scan/nsae091 (PMC11776713; doi:10.1093/scan/nsae091)
Supplement: nsae091_Supp [file nsae091_supp.zip › nsae091_Supp/scan-23-219-File010.docx]

**Supplementary Tables**

**Table S1.** Activated regions for the contrast of (NegReg > PosReg) > (NegAtt > PosAtt) with a traditional threshold (*p* < 0.001, uncorrected at the peak level, and *p* < 0.05, FWE　corrected at the cluster level).

| **Anatomical label** | **side** |  | **MNI** |  | ***k*** | ***t* value** |
| --- | --- | --- | --- | --- | --- | --- |
|  |  | ***x*** | ***y*** | ***z*** |  |  |
| **(NegReg > PosReg) > (NegAtt > PosAtt)** | | | | | | |
| Cerebellum | L | -45 | -58 | -25 | 338 | 5.16 |
| Inferior occipital cortex | R | 30 | -88 | -4 | 121 | 4.77 |
| MPFC/pre-SMA | R | 15 | 14 | 65 | 69 | 4.61 |
| **(PosReg > NegReg) > (PosAtt > NegAtt)** | | | | | | |
| n.s. |  |  |  |  |  |  |

*p* < 0.05, family-wise error corrected for multiple comparisons at the cluster level. Cluster sizes (*k*) and voxel-level *t* values are shown. MNI, Montreal Neurological Institute; n.s., not significant; R, right; L, left; MPFC, medial prefrontal cortex; pre-SMA, pre-supplementary motor area.

**Table S2.** Activated regions for comparisons between the Reg and Att conditions.

| **Anatomical label** | **side** |  | **MNI** |  | ***k*** | ***t* value** |
| --- | --- | --- | --- | --- | --- | --- |
|  |  | ***x*** | ***y*** | ***z*** |  |  |
| **PosReg > PosAtt** | | | | | | |
| Angular gyrus | L | -48 | -67 | 41 | 97 | 5.01 |
| Precuneus | L | -9 | -58 | 29 | 60 | 3.93 |
| **NegReg > NegAtt** | | | | | | |
| Supramarginal gyrus | L | -60 | -52 | 32 | 522 | 6.27 |
| Middle frontal gyrus | L | -39 | 26 | 38 | 924 | 5.53 |
| Cerebellum | R | 27 | -79 | -34 | 236 | 5.49 |
| Precuneus | L | -12 | -52 | 35 | 197 | 4.83 |
| Cerebellum | L | -30 | -79 | -34 | 75 | 4.56 |
| Superior frontal gyrus | R | 21 | 44 | 32 | 96 | 4.24 |
| **(PosReg + NegReg) > (PosAtt + NegAtt)** | | | | | | |
| Angular gyrus | L | -51 | -67 | 35 | 428 | 7.33 |
| Precuneus | L | -9 | -52 | 35 | 353 | 5.24 |
| Cerebellum | R | 39 | -58 | -43 | 159 | 5.36 |
| Superior frontal gyrus | L | -21 | 47 | 41 | 203 | 4.89 |
| Anterior cingulate gyrus | L | -3 | 26 | -4 | 66 | 4.41 |
| **PosAtt > PosReg** | | | | | | |
| Inferior temporal gyrus | R | 51 | -55 | -10 | 1816 | 6.80 |
| Inferior temporal gyrus | L | -45 | -64 | -13 | 1894 | 6.33 |
| Precentral gyrus | R | 51 | 11 | 29 | 121 | 5.25 |
| Precentral gyrus | L | -48 | 5 | 32 | 150 | 5.19 |
| Middle frontal gyrus | R | 30 | 5 | 59 | 96 | 4.96 |
| Precentral gyrus | L | -39 | -10 | 56 | 104 | 4.16 |
| **NegAtt > NegReg** | | | | | | |
| Precentral gyrus | L | -45 | 2 | 26 | 73 | 5.13 |
| Supramarginal gyrus | R | 45 | -31 | 44 | 420 | 4.81 |
| Inferior occipital gyrus | L | -42 | -64 | -7 | 72 | 4.48 |
| Middle occipital gyrus | L | -30 | -85 | 11 | 97 | 4.44 |
| Inferior temporal gyrus | R | 51 | -52 | -10 | 94 | 4.34 |
| **(PosAtt + NegAtt) > (PosReg + NegReg)** | | | | | | |
| Inferior temporal gyrus | R | 51 | -55 | -10 | 2253 | 7.74 |
| Inferior occipital gyrus | L | -42 | -67 | -7 | 1915 | 7.32 |
| Cerebellum | L | -18 | -70 | -49 | 268 | 6.58 |
| Middle frontal gyrus | R | 27 | -1 | 47 | 126 | 4.97 |

*p* < 0.05, family-wise error corrected for multiple comparisons at the cluster level. Cluster sizes (*k*) and voxel-level *t* values are shown. MNI, Montreal Neurological Institute; n.s., not significant; R, right; L, left.

**Table S3.** Results of the unpaired *t*-tests for sex differences in the fMRI activation.

| Functional region of interest | *t*-value | *p*-value |
| --- | --- | --- |
| L aIns | 0.61 | 0.54 |
| R aIns/TP | 0.85 | 0.39 |
| L SMG | 1.36 | 0.18 |
| pre-SMA (R SFG) | 0.85 | 0.40 |
| L Cerebellum | 0.01 | 0.98 |
| R Cerebellum | 0.43 | 0.66 |
| ACC/MPFC (L MSFG) | 0.80 | 0.42 |

R, right; L, left; aIns, anterior insula; TP, temporal pole; SMG, supramarginal gyrus; pre-SMA, pre-supplementary motor area; ACC, anterior cingulate cortex; MPFC, medial prefrontal cortex.
