# Supplementary material for: Cerebellar contribution to emotion regulation and its association with medial frontal GABA level
Source: Soc Cogn Affect Neurosci. 2024 Dec 2;20(1):nsae091. doi: 10.1093/scan/nsae091 (PMC11776713; doi:10.1093/scan/nsae091)
Supplement: nsae091_Supp [file nsae091_supp.zip › nsae091_Supp/scan-23-219-File011.docx]

**Supplementary Figure Legends**

**Fig. S1.** An example of the region of interest (ROI) for ^1^H-MRS data acquisition. A mask ROI image for an individual was normalized and then overlaid on the brain template image.

**Fig. S2.** Right cerebellar overlap between the results from the main analysis (red regions) and the contrast of (NegReg > PosReg) > (NegAtt > PosAtt) with a liberal threshold (blue regions; *p* < 0.005, uncorrected at the peak level, and *p* < 0.05, FWE-corrected at the cluster level).

**Fig. S3.** The left parietal clusters that were activated for either the main or an additional fMRI analysis. The green area was significantly activated for the contrast of (NegReg/male > PosReg/male) > (NegReg/female > PosReg/female) in the group-level analysis with three factors (task conditions, emotional valences, and biological sex) (MNI coordinate = [-63, -31, 23]; cluster size = 63; *t*-value = 4.57). The blue area was found in the contrast of NegReg versus PosReg in the main analysis.
