# Supplementary figures and images for: Cerebellar contribution to emotion regulation and its association with medial frontal GABA level
Source: Soc Cogn Affect Neurosci. 2024 Dec 2;20(1):nsae091. doi: 10.1093/scan/nsae091 (PMC11776713; doi:10.1093/scan/nsae091)

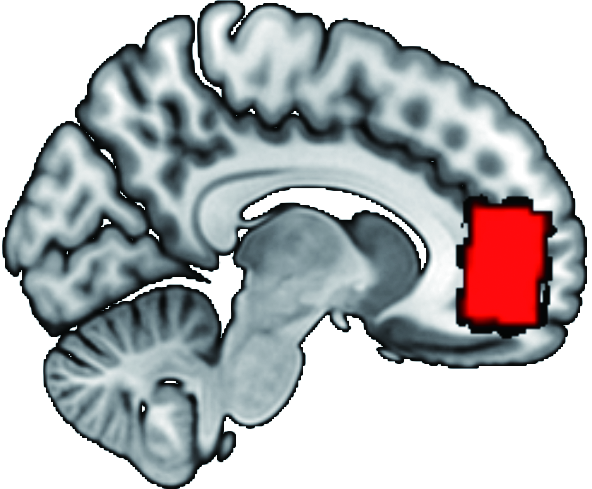

Supplement: nsae091_Supp [file nsae091_supp.zip › nsae091_Supp/scan-23-219-File012.tif]

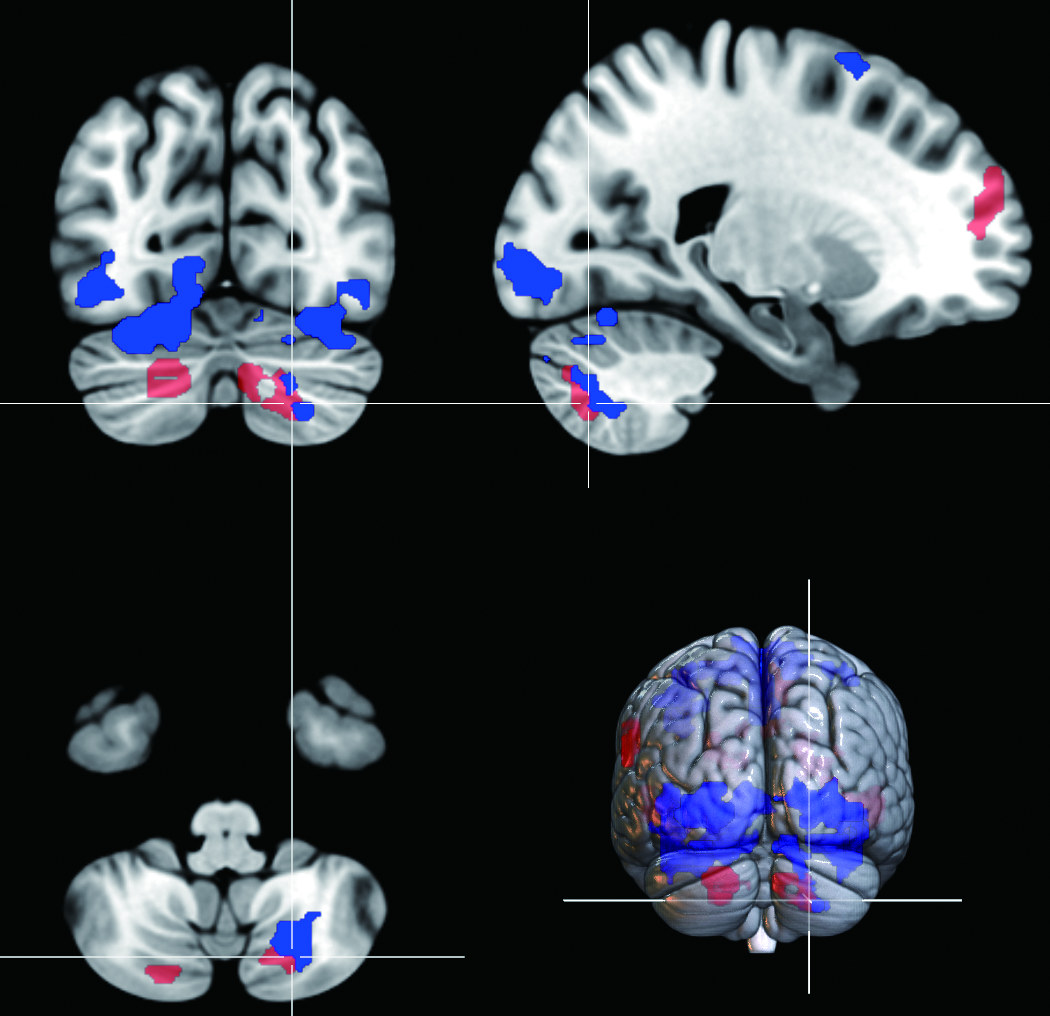

Supplement: nsae091_Supp [file nsae091_supp.zip › nsae091_Supp/scan-23-219-File013.tif]

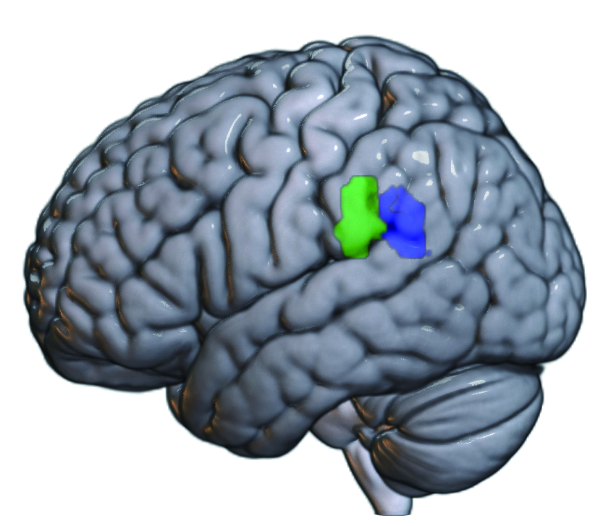

Supplement: nsae091_Supp [file nsae091_supp.zip › nsae091_Supp/scan-23-219-File014.tif]
